# Immediate Effects of Kinesio Taping on Rectus Abdominis Diastasis in Postpartum Women—Preliminary Report

**DOI:** 10.3390/jcm10215043

**Published:** 2021-10-28

**Authors:** Lucyna Ptaszkowska, Justyna Gorecka, Malgorzata Paprocka-Borowicz, Karolina Walewicz, Slawomir Jarzab, Marta Majewska-Pulsakowska, Joanna Gorka-Dynysiewicz, Anna Jenczura, Kuba Ptaszkowski

**Affiliations:** 1 Institute of Health Science, University of Opole, Katowicka 68, 45-060 Opole, Poland; lucyna.ptaszkowska@uni.opole.pl (L.P.); karolina.walewicz@uni.opole.pl (K.W.); anna.jenczura@uni.opole.pl (A.J.); 2 Department of Physiotherapy, Wroclaw Medical University, Grunwaldzka 2, 50-355 Wroclaw, Poland; justyna.gorecki@gmail.com; 3 Department of Clinical Biomechanics and Physiotherapy in Motor System Disorders, Faculty of Health Science, Wroclaw Medical University, Grunwaldzka 2, 50-355 Wroclaw, Poland; slawomir.jarzab@umed.wroc.pl (S.J.); marta.majewska-pulsakowska@umed.wroc.pl (M.M.-P.); kuba.ptaszkowski@umed.wroc.pl (K.P.); 4 Department of Pharmaceutical Biochemistry, Wroclaw Medical University, Borowska 211 A, 50-556 Wroclaw, Poland; joanna.gorka-dynysiewicz@umed.wroc.pl

**Keywords:** rectus abdominis diastasis, kinesio taping, postpartum women, surface electromyography

## Abstract

Background: Rectus abdominis diastasis (RAD) is an excessive divarication of the rectus abdominis muscle with concurrent stretching and thinning of the linea alba, which occurs due to mechanical and functional disturbances in the anterior abdominal wall and the whole body. The primary objective of this study is a palpation assessment of RAD in postpartum women before and after the application of KT tapes and a subsequent comparison of the results with those from a sham intervention group. Methods: A randomized clinical trial was conducted in the Physical Therapy Department at Wroclaw Medical University. The participants were randomly assigned to one of two groups: the KT group (intervention), in which KT tapes were applied (48 h intervention) and the sham KT group (control, sham intervention), in which non-stretch tapes were used (cloth surgical tape, 48 h intervention). In all participants, a palpation assessment of RAD was conducted and the inter-recti distance was measured using a digital caliper at three sites: at the umbilicus and 4.5 cm above and below it. Measurements were taken before and after the intervention. Results: The gathered results show a statistically significant reduction in rectus abdominis diastasis at each of the observed sites after the application of KT tapes in the intervention group (*p* < 0.05). In the intergroup comparison, a statistically significantly lower RAD (at umbilicus) was found after the intervention (*p* = 0.005) in KT group. Conclusions: the application of KT tapes using the corrective technique can contribute to reducing RAD in women up to 12 months after delivery.

## 1. Introduction

Rectus abdominis diastasis (RAD) is an excessive divarication of the rectus abdominis muscle with concurrent stretching and thinning of the linea alba, which occurs due to mechanical and functional disturbances in the anterior abdominal wall and the whole body. Due to the ontogenic development of the linea alba, RAD can manifest differently in individual patients as well as in different sections of the linea alba [1,2,3]. A separation of the heads of the rectus abdominis muscle of more than 2 cm is considered pathological. RAD can affect both women and men of any age [3,4,5,6]. However, it is most often diagnosed in pregnant and postpartum women, due to the changes occurring in a woman’s body during pregnancy [1,7,8]. RAD can occur in infants, people with abdominal obesity and those whose jobs involve heavy lifting [2,5,8]. The most common symptom of RAD is (conical) bulging along the midline of the abdomen—which becomes more prominent while abdominal muscles contract—together with a visible depression in the linea alba [1,2,3,5]. 

Statistically, 24 in 40 women require rehabilitation due to RAD. The most popular form of rehabilitation involves exercises that strengthen abdominal muscles. However, when performed in the incorrect order, they can worsen the condition [3,5]. Recently, Kinesio taping (KT)—applying special tapes directly to the skin—has been gaining in popularity. In the case of RAD, application of such tapes can act as supportive therapy and help protect the linea alba against further stretching [9,10,11,12].

The use of KT in reducing RAD in women after childbirth has not been thoroughly investigated so far. Based on the literature [9,10], we can find only indications of the beneficial effect of using KT or KT and exercises in reducing RAD. It should be emphasized that the scientific evidence presented so far contains numerous methodological limitations. It seems that further studies evaluating these relationships should be conducted. The primary objective of this study was to determine the immediate effect of KT application on RAD. The expectation is that RAD will decrease due to the application of KT tapes. A secondary objective is an electromyographic assessment of the effect of Kinesio taping on the rectus abdominis. The hypothesis is that the bioelectrical activity of the muscles will increase as a result of the application of KT tapes.

## 2. Methods

A randomized clinical trial was conducted in the Physical Therapy Department at Wroclaw Medical University. The project was approved by the Institutional Review Board at Wroclaw Medical University with the number KB–43/2018 dated 06.02.2018. This study was registered as a Clinical Trial with the identifier number ISRCTN36874773. The target group of the study consisted of women with diagnosed RAD from 6 weeks to 12 months after childbirth.

All participants were assessed according to the inclusion and exclusion criteria established for the study. The inclusion criteria encompassed: RAD > 2 cm at least at one of three sites, postnatal period > 6 weeks and <12 months, BMI < 30, and consent to participate in the trial. The exclusion criteria consisted of: multiple pregnancy, cesarean delivery, other surgeries in the abdominal area, and lack of consent to participate in the trial.

The participants were randomly assigned to one of two groups: a KT group (intervention), in which KT tapes were applied, and a sham KT group (control, sham intervention), in which non-stretch tapes were used (cloth surgical tape). Randomization was carried out using computer-generated random numbers (simple randomization). The participants were randomly assigned to groups in a 1:1 ratio. The protocol for every visit of a participant included an interview, a briefing about the test procedures and the purpose of the measurements, obtaining the consent of participation in the trial, completing a survey questionnaire, preparing the participant for the testing, and taking electromyographic measurements of the rectus abdominis muscle. In all participants, a palpation assessment of RAD was conducted and the inter-recti distance was measured using a digital caliper at three sites: at the umbilicus and 4.5 cm above and below it. The measurements were taken with the participant in the supine position with lower limbs bent in the hip and knee joints, and the trunk flexed forward. 

**Figure 1:**
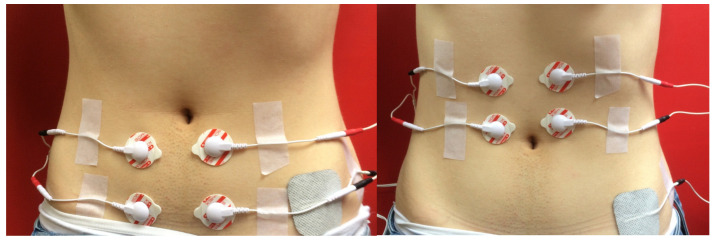
sEMG electrodes placement.

**Figure 2:**
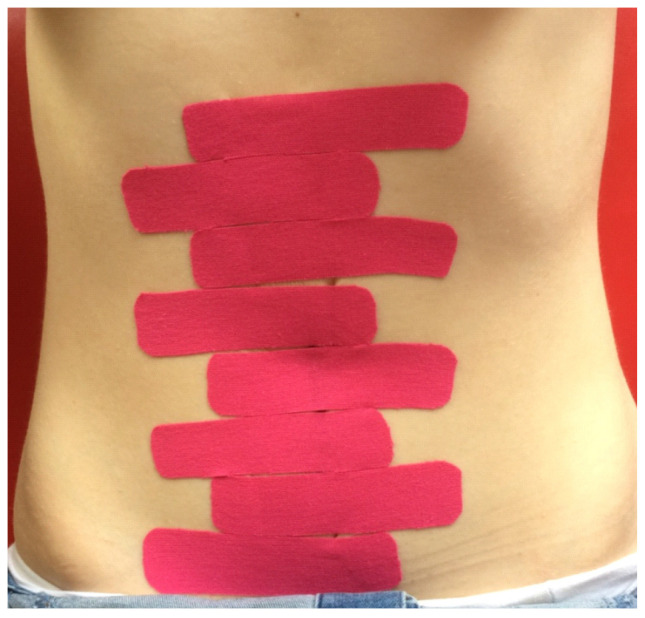
The KT application.

Subsequently, surface electromyography (sEMG) of the rectus abdominis above and below the umbilicus was conducted using the MyoPlus 4 Pro electromyograph (Verity Medical Ltd., Romsey, UK), with the left and right sides of the abdomen measured separately. Two working electrodes were placed on each belly of the rectus abdominis, and a reference electrode was placed at the anterior superior iliac spine (Figure 1). Measurements were taken for resting activity, functional activity, and isometric contraction. The procedure was performed twice, first for the muscle section above the umbilicus, and then for the section below. The next stage was the application of KT tapes (K-Active Tape-a flexible, adhesive tape consisting of a cotton fabric and an acrylic adhesive layer; elasticity 130–140%, Nitto Denko K-Active^®^ Tape, Nitto Denko Tape Materials Corporation Ltd., Osaka, Japan) in the intervention group, using the corrective (mechanical) technique with a 75–100% tension range. In the control group, non-stretch tapes were used (cloth surgical tape). The tapes were placed perpendicularly to the rectus abdominis in the form of 2.5 cm-wide strips along the entire length of the muscle, crossing the midline of the linea alba (Figure 2). The application period was 48 h. After that time, the tapes were removed and the measurements for the width of RAD and sEMG were repeated. 

Based on the results of the preliminary study (unpublished, *n* = 5 in each group), the sample size was estimated (Statistica 13.3; TIBCO Software Inc.; Palo Alto, CA, USA). Means and standard deviations of the differences between the results of inter-recti distance (at the umbilicus) before and after the intervention in the two groups were used in the analysis for estimating the sample size. The estimated sample size for a two-sample unpaired-means test (unpaired t-test). Parameters: mean the difference in KT group was 1.1 cm (SD = 0.7 cm); mean in sham KT group was 0.2 cm (SD = 0.4 cm); the alpha level was set at 0.05, and the power of the test at 0.8. It also assumed no correlation of evaluated variables and adopted a 2-sided null hypothesis. Based on the parameters, the estimated sample size obtained a minimum of 11 people in each group.

The statistical analysis was performed using the program Statistica 13.3 (TIBCO Software Inc.; Palo Alto, CA, USA).). For the measurable variables, the mean, median minimum, and maximum value quartiles were calculated. All tested quantitative variables were tested with the Shapiro–Wilk test to determine the type of distribution. The reliability and repeatability of these measurements were assessed using an intraclass correlation coefficient (ICC). In each case, r > = 0.90. The comparisons of results between the groups were performed using the nonparametric U-Mann–Whitney test. The Wilcoxon signed-rank test was used in the analysis comparing the results before and after the tape application in both groups. The significance level of α = 0.05 was adopted for all comparisons.

## 3. Results

**Figure 3:**
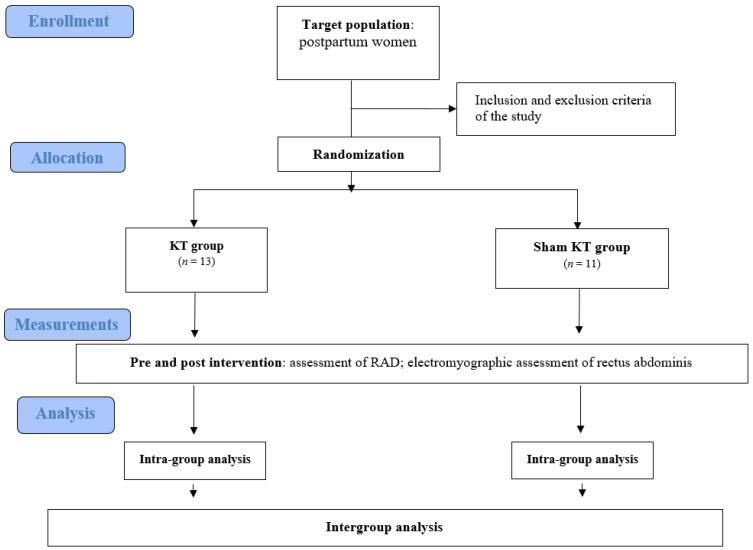
The flow of the participants at each stage of the project.

The participants of the trial included 24 postpartum women aged 18–38 with diagnosed rectus abdominis diastasis above 2 cm at least at one of three sites. Four women were excluded from the study based on the inclusion and exclusion criteria: two women had previously had other surgeries in the abdominal area, one woman had RAD < 2 cm in all of three sites, and one had had multiple pregnancies. The participants were randomly divided into two groups for comparison: the KT group (13 people) and the sham KT group (11 people). Figure 3 presents the flow of the patients at each stage of the project.

Table 1 shows the characteristics of both groups with respect to the age, height, and weight of the participants. The mean age in the KT group was 27.5, and in the sham KT group, 28. The mean BMI in the intervention group was 22.1 kg/m^2^ and in the control group 21.3 kg/m^2^ (Table 1). Table 1 also includes data on the length of the last delivery, the birth weight of the child, and the time between the delivery and the trial for all participants. The mean length of the last delivery in the KT group was 37.2 min and in the sham KT group 30 min (these data pertain to the last stage of labor). The mean time between the delivery and the trial was 27 weeks in the KT group and 32 weeks in the sham KT group (Table 1). 

In terms of the effects of KT on rectus abdominis diastasis, the gathered results show a statistically significant reduction in rectus abdominis diastasis at each of the observed sites after the application of KT tapes in the intervention group (*p* < 0.05) (Table 2). In the control group, no statistically significant differences were found at any of the studied sites before or after the application of non-stretch tapes (*p* > 0.05) (Table 2). In the intergroup comparison, a statistically significantly lower RAD (at umbilicus) was found after the intervention (*p* = 0.005) in KT group.

In terms of the comparison of the sEMG measurements before and after the application of KT, the tables below compare the measurements of the bioelectric activity in the rectus abdominis muscle before and after the application of KT tapes in the KT group (Table 3) and non-stretch tapes in the sham KT group (Table 4). The gathered results do not show statistically significant differences in the sEMG measurements of the rectus abdominis above the umbilicus and below the umbilicus before or after the application of KT tapes in the KT group. There were also no statistically significant differences in the sEMG measurements of the rectus abdominis muscle above the umbilicus before and after the application of non-stretch surgical tapes in the sham KT group. However, statistically, significant differences were observed in the sEMG measurements of the rectus abdominis below the umbilicus before and after the application of non-stretch surgical tapes in the sham KT group (functional sEMG activity—left and right side and during isometric contraction—right site). In the intergroup comparison, only statistically significant differences in the measurement of sEMG during isometric contraction above the umbilicus on the left (*p* = 0.024) and right side (*p* = 0.037) were found.

## 4. Discussion

The abdominal muscles fulfill many important roles, such as maintaining good posture, breathing, ensuring continence, and bending the trunk [4,5,13]. The main goal of physical therapy is restoring the optimal function, mobility, and flexibility of structures such as the diaphragm, chest, and pelvis. Strengthening the weakened muscles occurs only in subsequent stages. There is no universal training program for RAD, as every case varies and has different causes and consequences [1,3,7,8,14,15,16]. 

Kinesio taping is a non-invasive physical therapy method that involves adhering special tapes directly to the skin using the appropriate technique in the problem area. It is based on employing techniques that support the body’s self-healing properties. In the case of RAD, it is the physical therapist who should decide whether to implement this method. The type of application, its direction, and the tension of the tapes depend on the aim and stage of the rehabilitation. The aims of Kinesio taping in rectus abdominis diastasis include: protecting the linea alba; protecting the hernia (if applicable); supporting tissue regeneration; and strengthening the effects of physical therapy [5,9,10,11].

Postpartum women recover at different paces. Typically, the postnatal period lasts from 6 to 8 weeks, and during that time, the woman should regain the condition from before the pregnancy and delivery. RAD can affect 27% of pregnant women in the second trimester and 66% of those in the third trimester. Mota et al. [4] claim that the issue can occur in as many as 100% of pregnant women. Analysis of Brusch’s [3] study results show that, in 40 women studied in the postnatal period, 60% required rehabilitation due to RAD. Thabet [17] proposes an exercise program for stabilizing deep core muscles. In their study, they examined 40 postpartum women, whom they divided into two groups: the first group performed traditional exercises that strengthen the abdominal muscles as well as exercises for deep core stability, and the second group only performed the traditional exercises that strengthen the abdominal muscles. The therapy lasted 8 weeks with a frequency of 3 exercise sessions per week. A statistically significant reduction in RAD (*p* < 0.0001) was observed in the first group. Kamel et al. [18] conducted a study on 60 women, 2 months after childbirth, whom they divided into two groups. The first group performed exercises for abdominal muscles and electrostimulation of the rectus abdominis and the second group only performed the strengthening exercises. A significant improvement (*p* < 0.05) in RAD reduction was shown in both groups, with the biggest advantage being found in the first group.

Benjamin et al. [2] examined the relation between RAD and low back pain, incontinence, and pelvic organ prolapse, as well as between abdominal muscle strength and quality of life. A total of 2242 women with diagnosed RAD participated in the study. The study did not find a significant connection between the presence of RAD and lumbo-pelvic pain or incontinence. However, it demonstrated an association between the presence of RAD and pelvic organ prolapse.

Benjamin et al. [1] conducted a meta-analysis of the literature available in EMBASE, Medline, CINAHL, PUBMED, AMED, and PEDro. The objective was to determine whether exercise can prevent or reduce RAD. Benjamin et al. analyzed 8 studies involving 336 pregnant and postpartum women. The methods ranged from case studies to randomized control trials. All therapeutic interventions involved exercises strengthening abdominal muscles. The studies showed that physical activity before delivery reduced the risk of RAD by 35%.

The sEMG measurement was an additional test in this study. In the control group, bioelectrical activity in some cases had decreased. Our hypothesis was that activity after the intervention would increase, contrasting with the fact there was a decrease in bioelectric activity. Further research would be worthwhile to verify the observed result. It was observed that no statistically significant reduction in RAD was observed for the control group. On the other hand, there was a decrease in bioelectrical activity in some cases. This can be explained as a normalization of tension, but it is hard to find a confirmation of our supposition.

The limitations of the present study include a low number of participants in the compared groups and a short period of tape application. The subjective assessment of the size of the diastasis also worked to the study’s disadvantage. Ultrasonography of the abdominal wall would be a more meaningful test, as it would allow more precise measurement of RAD. Another limitation of the study is the method of measuring the functional bioelectric activity of the rectus abdominis, as it is possible that, during the second visit, the participant might have used a different amount of power for trunk flexion “crunch” than in the first measurement. Furthermore, in the measurement of the functional bioelectric activity of the rectus abdominis below the umbilicus, the gathered results might be unreliable due to the lack of activity of the muscle at the insertion (the lower part of the trunk). Additionally, no follow-up examination of the result of the KT tape application was performed; therefore, the longevity of its therapeutic effect is unknown. More research into the most effective methods of physical therapy and supportive treatment for RAD in postpartum women is necessary.

## 5. Conclusions

Our data suggest that the application of KT tapes using the corrective technique can contribute to reducing RAD in women up to 12 months after delivery. However, it seems that this is not clearly associated with an increase in the bioelectrical activity of the rectus abdominis muscles. It is necessary to conduct studies on a larger population, considering additional objective RAD assessments.

## Figures and Tables

**Table 1:** Comparison of the demographic and clinical characteristics of participants between the two groups.

	KT Group (*n* = 13)					Sham KT Group (*n* = 11)					*p*-Value *
	Mean	Me	Min	Max	SD	Mean	Me	Min	Max	SD	
Age	27.5	27	20	38	5.8	27.6	28	18	34	4.4	0.75
Height [m]	1.7	1.7	1.6	1.8	0.1	1.7	1.7	1.6	1.8	0.1	0.83
Body weight [kg]	61.4	62	50	70	6.4	61.9	60	52	60	8.7	0.87
BMI [kg/m^2^]	22.1	22	19.5	25.4	1.9	22.1	21.3	19.3	25.7	2.9	0.64
Birth weight of the child [g]	3461.9	3500	2825	4150	419.9	3405.5	3500	2500	3950	400.6	0.95
Length of the last delivery [min.]	37.2	30	10	90	24.7	26.8	30	10	50	13.1	0.60
Time since the last delivery [weeks]	27.6	25	7	50	15.8	31.7	32	6	52	14.5	0.74

*n*—number of participants; Me—median; Min—minimum value; Max—maximum value; SD—standard deviation * U-Mann–Whiney test.

**Table 2:** The comparison of RAD before and after intervention in both groups.

	Before					After					*p*-Value *
	Mean	Median	Min.	Max.	SD	Mean	Median	Min.	Max.	SD	
	**KT group (*n* = 13)**										
At umbilicus	1.9	2.2	1.3	2.5	0.5	1.7	1.8	1.2	2.2	0.3	0.003
Above umbilicus	1.6	1.6	0.6	2.4	0.6	1.2	1.2	0.5	2.2	0.5	0.002
Below umbilicus	1.1	1.3	0.02	1.8	0.6	0.8	1.0	0.0	1.4	0.5	0.008
	**Sham KT group (*n* = 11)**										
At umbilicus	2.1	2.2	1.0	2.5	0.4	2.1	2.2	1.3	2.5	0.4	0.55
Above umbilicus	1.6	1.5	1.2	2.2	0.3	1.6	1.4	1.0	2.3	0.4	0.35
Below umbilicus	1.1	1.2	0.01	2.2	0.8	1.1	1.1	0.01	2.1	0.7	0.12

*n*—number of participants; Me—median; Min—minimum value; Max—maximum value; SD—standard deviation * Wilcoxon test.

**Table 3:** sEMG measurements before and after the KT application in the intervention group.

sEMG Activity	Before					After					*p*-Value *
	Mean	Median	Min.	Max.	SD	Mean	Median	Min.	Max.	SD	
	**Right side above umbilicus**										
Resting	1.2	1.0	0.3	4.0	0.9	1.3	1.1	0.5	5.1	1.2	0.76
Functional	15.5	13.4	1.2	39	13	22.3	21.2	2.1	41.4	12.8	0.65
During isometric contraction	21.0	19.6	3.5	54.7	16.4	19.7	19.2	1.6	41.2	12.4	0.42
	**Right side below umbilicus**										
Resting	1.6	0.9	0.3	7.3	2.9	1.3	1.1	0.4	3.6	1.1	0.26
Functional	5.8	3.1	0.7	25.5	6.8	5.4	4.9	0.8	20.1	5.5	0.55
During isometric contraction	7.2	2.5	0.6	23.8	8.4	6.3	2.9	0.8	31.2	7.4	0.39
	**Left side above umbilicus**										
Resting	0.7	0.6	0.2	1.6	0.4	0.9	0.6	0.4	2.5	0.6	0.17
Functional	21.6	21.6	1.1	58.5	16.8	21.8	24.2	7.5	41.7	10.0	0.70
During isometric contraction	21.4	20.5	0.8	49.4	16.1	19.3	20.3	6.6	34.2	8.9	0.97
	**Left side below umbilicus**										
Resting	0.9	0.7	0.3	3.2	0.8	0.8	0.6	0.3	2.0	0.4	0.96
Functional	6.6	7.0	0.7	12.6	3.6	7.4	5.9	1.5	18.6	4.8	0.55
During isometric contraction	7.1	7.3	1.2	17.7	4.5	7.8	5.5	2.2	18.4	4.8	0.34

*n*—number of participants; Me—median; Min—minimum value; Max—maximum value; SD—standard deviation * Wilcoxon test.

**Table 4:** sEMG measurements before and after the application of non-stretch tapes in the sham KT group.

sEMG Activity	Before					After					*p*-Value *
	Mean	Median	Min.	Max.	SD	Mean	Median	Min.	Max.	SD	
	**Right side above umbilicus**										
Resting	0.7	0.7	0.4	1.0	0.2	0.8	0.5	0.3	1.7	0.5	0.79
Functional	16.7	17.0	4.4	29.1	8.5	14.6	12.0	7.0	30.1	8.1	0.25
During isometric contraction	10.8	11.4	4.7	22.9	5.1	10.4	11.1	5.3	17.2	3.4	0.80
	**Right side below umbilicus**										
Resting	0.8	0.5	0.3	2.2	0.6	0.7	0.4	0.3	2.4	0.6	0.21
Functional	8.5	6.4	2.5	17.7	4.4	6.4	5.6	1.8	11.7	3.4	0.005
During isometric contraction	7.7	6.7	2.2	17.3	4.7	5.5	5.5	1.7	10.5	2.9	0.023
	**Left side above umbilicus**										
Resting	0.6	0.5	0.4	1.3	0.3	1.0	0.9	0.3	2.4	0.7	0.28
Functional	19.5	14.1	2.1	36.9	10.9	16.6	14.1	3.7	35.9	8.8	0.37
During isometric contraction	13.8	11.0	2.4	31.7	8.1	11.6	11.2	3.7	18.3	4.2	0.21
	**Left side below umbilicus**										
Resting	0.7	0.5	0.3	1.6	0.4	1.2	1.0	0.3	2.3	0.7	0.051
Functional	8.3	7.1	3.3	18.0	4.4	4.2	2.6	1.4	9.1	2.9	0.006
During isometric contraction	7.5	6.1	2.2	22.1	5.6	4.4	2.8	1.5	9.9	3.1	0.11

*n*—number of participants; Me—median; Min—minimum value; Max—maximum value; SD—standard deviation * Wilcoxon test.

## Data Availability

Data available on request due to restrictions eg privacy or ethical.
